# Oral conditions and salivary analysis in HIV-uninfected subjects using preexposure prophylaxis

**DOI:** 10.4317/medoral.25140

**Published:** 2022-04-03

**Authors:** Nayara Macedo, Gabriela Baggio, Indiara Henn, Juliane Santos, Thiago Batista, Sérgio Ignácio, Paulo Souza, Luciana Azevedo-Alanis

**Affiliations:** 1 MSc, Graduate Program in Dentistry. School of Life Sciences, Pontifícia Universidade Católica do Paraná, Curitiba, PR, Brazil; 2 PhD, Graduate Program in Dentistry. School of Life Sciences, Pontifícia Universidade Católica do Paraná, PR, Brazil; 3 DDS, PhD. Graduate Program in Dentistry, School of Life Sciences, Pontifícia Universidade Católica do Paraná, Curitiba, PR, Brazil

## Abstract

**Background:**

New prevention strategies have been advocated to control the progression of HIV/AIDS, such as preexposure prophylaxis (PrEP). The aim of this study is to evaluate the potential changes in the oral and salivary conditions of HIV-uninfected subjects using PrEP.

**Material and Methods:**

Subjects were evaluated before beginning the medication (T0), at the first follow-up (T1), and at the second follow-up (T2). Xerostomia, presence of untreated cavitated caries, oral hygiene habits, taste, gingival and plaque index, stimulated salivary flow rate (SSFR), and salivary concentrations of calcium, glucose, urea, and total proteins were evaluated. Data obtained were analyzed using statistical tests (*p*<0.05).

**Results:**

Forty-seven participants (41 men; 6 women) were evaluated at T0. Thirty (28 men; 2 women) and 17 men were reassessed at T1 and T2, respectively. There was no difference between the SSFR and oral and salivary conditions between T0, T1, and T2 (*p*>0.05), except for the salivary calcium concentration, that increased at T2 compared to T1 (*p*=0.02). There was significant difference between taste and xerostomia at T1 (*p*=0.017), and the need to drink to swallow at T2 (*p*=0.015). There was significant correlation between the reported amount of saliva and taste (*p*=0.039, r=-0.378) at T1.

**Conclusions:**

The prolonged use of PrEP seems to be associated with reports of dry mouth and worsening of taste, possibly associated with increased salivary calcium concentration.

** Key words:**Antiretroviral agents, pre-exposure prophylaxis, saliva, oral health.

## Introduction

AIDS is a significant global public health problem. By the end of 2018, 37.9 million people worldwide had HIV infection. Besides, in that year, approximately 1.7 million new infections occurred, with 54% of these new infections affecting key populations and their sexual partners, such as people who use alcohol and other drugs, serodiscordant couples, transvestites and trans people, gays, men who have sex with men, sex workers, and people deprived of their liberty (1). Given that no single prevention method or approach can stop the HIV epidemic, some new methods and interventions have been adopted to reduce the risk of HIV infection, including the use of antiretroviral drugs, such as preexposure prophylaxis (PrEP) (2).

Randomized clinical studies, such as the Preexposure Prophylaxis Initiative (iPREX), showed the effectiveness of the daily oral administration of PrEP to reduce HIV-1 infection by more than 90% in men who have sex with men with good treatment adherence (2-4). Since 2015, the World Health Organization (WHO) recommended that healthy people at risk of exposure to HIV use PrEP (5). PrEP consists of the daily use of a Tablet composed of tenofovir disoproxil fumarate and emtricitabine (TDF/FTC,Truvada®) (3). In Brazil, since 2017, PrEP has been provided free of charge to individuals from key populations engaging in HIV risk behavior. As of early 2021, nearly 22,000 individuals were receiving PrEP through the Brazilian Public Health System (6).

Based on the increasing number of patients on HIV PrEP, knowing its risks and benefits is important from a public health perspective. Among the systemic side effects, the most common are headaches, nausea, and arthralgia (2,4), as well as abdominal pain, vomiting, and dizziness (7). These symptoms are more frequent within the first month of beginning PrEP and tend to cease within three months (7). Other systemic problems related to the continuous use of this medication have also been reported, such as decrease in bone mineral density and decline in renal function (8,9).

The local effects of PrEP on oral health are poorly understood. However, oral manifestations resulting from adverse effects of antiretroviral medications in the same class as tenofovir, such as lamivudine and zidovudine, have been described, including xerostomia that may or may not reflect hyposalivation, and dysregulation of the cell/proliferative cycle and differentiation pathways of the gingival epithelium, resulting in fragile tissue repair and increased epithelial proliferation (10,11). Therefore, given this gap in the knowledge of the adverse effects of PrEP, this study aimed to evaluate the potential changes in oral and salivary conditions in HIV-uninfected individuals using PrEP. The hypothesis of this study, not yet explored in the literature, was that the use of PrEP was associated with changes in the oral and salivary conditions of users.

## Material and Methods

This was an observational, prospective, and analytical study. The research project was approved by the Local Ethics Committees (number 3.227.397) and by the Municipal Health Department of Curitiba (number 3.303.788).

- Sample selection

The study population consisted of participants at risk of exposure to HIV, selected for PrEP at the Orientation and Counseling Center of the Municipal Health Department of Curitiba (Curitiba, south Brazil).

Male and female patients aged 18 and older who started PrEP between May and December 2019 were invited to participate in the study, constituting a convenience sample. Participants who had clinical, systemic, and oral diseases and/or conditions that contraindicated the intraoral examination, or who presented with oral and/or systemic conditions that could interfere with salivary flow (i.e. hypothyroidism, history of radiotherapy in the head and neck, and/or chemotherapy in the previous three months) were excluded (12). All patients agreed to participate in the study after reading, understanding, and signing a free and informed consent form.

The patients were evaluated before they started using PrEP (T0) and at two time points after starting the medication, T1 and T2, referring to the second and third time they were dispensed medication, respectively.

- Oral conditions - Clinical examination

The patient’s demographic data and medical history were obtained from the database of the Orientation and Counseling Center in Curitiba (1).

The presence of xerostomia was evaluated using the question, “Do you have a dry mouth sensation?”. If the answer was “yes,” then xerostomia was considered present. This assessment was complemented using the following questions: “How do you describe the amount of saliva in your mouth?”, “Do you have difficulty in swallowing food?” and “Do you need any drink to swallow your meals?” (12,13). The xerostomia questionnaire was applied at three time points (T0, T1, T2), and for the analysis of the results, each question was considered individually.

Taste was assessed based on the chemotherapy-induced taste alteration scale (CiTAS). In this study, the values of the second dimension “discomfort” and the final CiTAS were used. The second dimension assesses the presence of nausea or malaise, changes in the sense of smell, and difficulty in eating meat. Its value is obtained using the average scores of the answers to the six questions regarding the dimension and varies from 1 to 5. The final CiTAS is the sum of the values of each dimension, ranging from 4 to 20; the higher the value obtained, the greater the taste change (14).

The assessment of oral hygiene habits was performed only at the initial consultation (T0) using a questionnaire composed of the questions: “Do you brush your teeth?”, “How many times a day?”, “Do you use dental floss?”, "Do you feel like your teeth are mobile?" and "Have you ever sought dental treatment?". (Supplement 1)

Patients were asked about the possible changes felt after beginning PrEP. The question "Did you feel any changes after starting treatment?" covered both the changes felt in the mouth (dryness and bitter taste) and other parts of the body (malaise, nausea, vomiting, and diarrhea) and was performed at both T1 and T2.

Extraoral and intraoral examinations were conducted at three time points (T0, T1, T2). An examination of the oral mucosa and teeth was performed through inspection and palpation using wooden spatulas to assist visualization with ambient light and, when necessary, with a porTable flashlight. Any alteration of the oral mucosa and its location were registered following WHO guidelines for epidemiological surveys (15).

Caries experience was evaluated using the DMFT index (decayed, missing, and filled) for teeth. The DMFT information allowed us to explore the prevalence of untreated cavitated caries among the participants. Cavitated untreated caries refer to the deterioration of a tooth's surface that has not been treated or filled. This was a binary variable categorized as "yes" or "no", indicating the presence or absence of untreated cavitated carious lesions, regardless of the number of affected teeth (16). The diagnostic criteria followed those proposed by WHO (17).

The gingival index (GI) and plaque index (PI) were applied using the diagnostic criteria proposed by Löe and Silness to determine the degree of gingival inflammation and quantify dental plaque, respectively (18,19). For the data analysis, the highest frequency of GI and PI per tooth was considered per patient. The GI and PI were analyzed according to the degree of severity (absence of plaque and inflammation: grade 0 and presence: grades 1, 2, and 3) (15).

- Saliva collection

Salivary collections respected the circadian rhythm and hormonal periods. The participants were asked about their last meal before collection; a minimum interval of 1 hour between their last meal and saliva collection was considered. The masticatory method was used to collect stimulated saliva at T0, T1, and T2. Participants were asked to chew a piece of rubber dike of a standard size (1.5 cm) tied to dental floss to prevent swallowing or aspiration continuously for 6 min. All saliva produced during the first minute of stimulation was neglected, allowing the participant to swallow it. During the next 5 min, participants chewed a piece of rubber dam and expelled the saliva inside a sterilized universal collection pot previously labeled and weighed, with guidance to avoid contamination when handling the pot. Once the saliva collection was finished, the collection jars with the salivary samples were immediately placed in a thermal box with ice (17). The jars containing the samples were stored at -20°C.

- Salivary analysis

Sialometry

The stimulated salivary flow rate (SSFR) was obtained at T0, T1, and T2 using the gravimetric method (20).

Sialochemistry

Salivary total protein, urea, glucose, and calcium concentrations in T0, T1, and T2 were evaluated. Before submitting salivary samples to biochemical tests, they were centrifuged for 5 min at 1,500 rpm (21).

The equipment used for the sialochemical analysis was a Cobas Mira Plus (Roche Diagnostic Systems, Basel, Switzerland). For the analyses, Bioclin reagents (Bioclin/Quibasa, Belo Horizonte, Brazil) were used, and the manufacturer's recommendations were followed.

To measure the salivary concentrations of total proteins, glucose, urea and calcium, their respective methodologies, sensitivity, and linearity of the method are described below: colorimetric biuret, 0.043 g/dL and 12 g/dL; colorimetric enzyme GOD-hydrogen peroxide, 1.31 mg/dL and 500 mg/dL; fixed-time kinetics, 1.51 mg/dL and 300 mg/dL; endpoint colorimetric, Arsenazo III, 0.074 mg/dL and 20 mg/dL. Samples that showed values above linearity were diluted for analysis. Dilutions were made according to the manufacturer's instructions. In the samples that presented values below the sensitivity, the test's sensitivity value, indicated by the manufacturer, was considered.

- Statistical analysis

The data obtained were tabulated, and an analysis was performed using SPSS version 24.0 (IBM Software, New York, NY). For normality analyses, the Kolmogorov-Smirnov test was used, and the Levene test was used for equality of variances. Other tests used were the Mann-Whitney U test, Kruskal-Wallis non-parametric test, McNemar test, and Friedman non-parametric test for paired tests. Comparisons over time were performed using the pairwise method, and Spearman's correlation coefficient was used for correlations. A significance level of *p*<0.05 was considered.

## Results

- Demographic data

Forty-seven participants who started using PrEP between May and December 2019 were included in the analysis. Demographic characteristics of the sample are described in Table 1.

**Figure 1:**
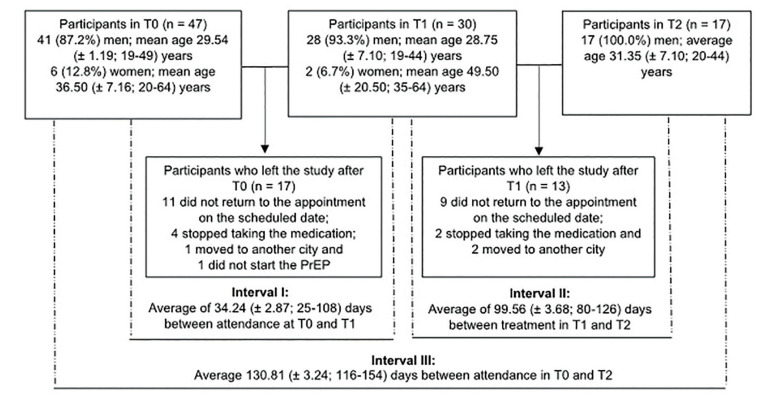
Follow-up of the participants at the three time points (T0, T1 and T2) and the respective sample losses. Interval I refers to the days elapsed from the initial consultation at T0 to the next consultation, T1. Interval II refers to the days elapsed from the consultation at T1 to the next consultation, T2. Interval III refers to the days elapsed from the consultation at T0 until the final consultation, T2.

Of the 47 participants who enrolled in the study at T0, 41 (87.2%) were male, and six (12.8%) were female. Fig. 1 illustrates the patient follow-up flowchart.

**Table 1:**
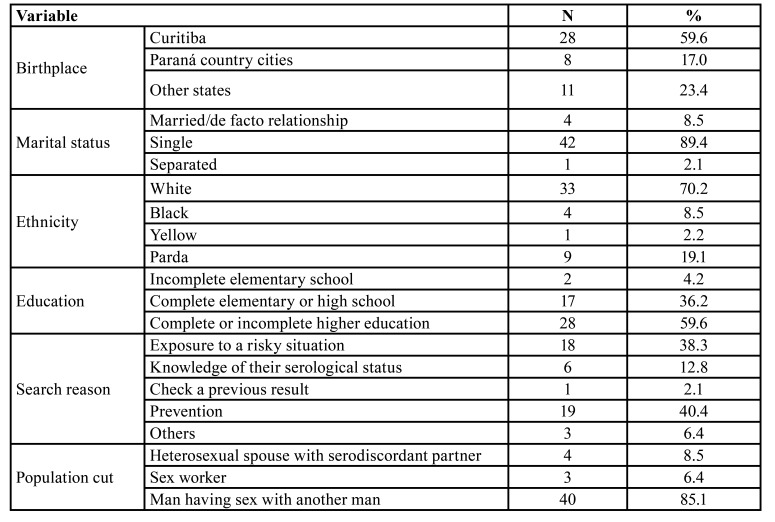
Demographic characteristics of patients using PrEP from the Guidance and Counseling Center of the Municipal Health Department of Curitiba between May and December 2019 (n = 47).

- Oral clinical examination

The oral mucosal changes and the total number of changes present in the participants at the three time points are described in Table 2. In addition to the changes that persisted over time (linea alba and fissured tongue), dryness of the lips and oral mucosa accounted for four (22.2%) of the 18 and four (33.4%) of the 15 changes observed in the intraoral examination at T1 and T2, respectively.

In T0, out of a total of 47 participants, eight (17%) presented with untreated cavitated caries. In T1 and T2, these numbers were four (13.3%) and one (5.9%) out of a total of 30 and 17 participants, respectively. Among the participants who had untreated cavities at T0 and were followed-up at T1 and T2, it was observed that caries persisted, indicating a lack of dental treatment.

At T0, the amount of saliva collected was insufficient that is, low quantity in three patients (n=44). Of these three patients, only one followed the treatment, resulting in a difference of one patient less for the salivary analyses in T1 (n=29) and T2 (n=16). When the variables GI, PI, taste, and frequency of response to questions about xerostomia (n=17) and SSFR (n=16) were compared between the three time points (T0, T1, T2), there was no significant difference (*p*>0.05; Table 3).

A total of 21 (65.6%) of the 30 and 11 (64.7%) of the 17 participants answered positively to the question, "Did you feel any changes after starting treatment?" at T1 and T2, respectively. There was no significant difference in the answers to this question at T2 compared to T1 (*p*>0.05). Of the 11 participants who experienced any changes after starting treatment at T2, four (36.4%) had already reported experiencing changes at T1 (polydipsia, malaise, dry mouth, and dry lips), and six (54.5%) patients reported having experienced some change only at T2 (malaise, polydipsia, dry mouth, and increased appetite). (Supplement 2)

- Salivary analysis

The average interval between the last meal the patient had and saliva collection was 206.6 ± 32.6 min at T0, 175.5 ± 40.4 min at T1, and 198.0 ± 26.4 min at T2 (*p*>0.05).

The values of the SSFR and salivary concentrations of calcium, glucose, total proteins, and urea were compared between the three time points for participants who used PrEP throughout the study (n=16). There was a significant increase in the salivary calcium concentration values at T2 compared to T1 (*p*=0.02; Table 4).

At T1, there was a significant difference between the average score of the variable taste (discomfort) in relation to xerostomia. Those patients who had a dry mouth sensation presented greater discomfort for taste (*p*=0.017). At T1, there was a significant difference between the mean value of SSFR and xerostomia, and those who had a dry mouth sensation had a lower SSFR (*p*=0.041). At T1, there was a significant difference between the mean value of calcium concentration and xerostomia, and those who reported dry mouth had a higher salivary calcium concentration (*p*=0.034). At T1, there was also a significant difference between the average taste score (final CiTAS) and xerostomia, with those who reported feeling dry mouth had a higher score for taste (final CiTAS; *p*=0.013).

**Table 2:**
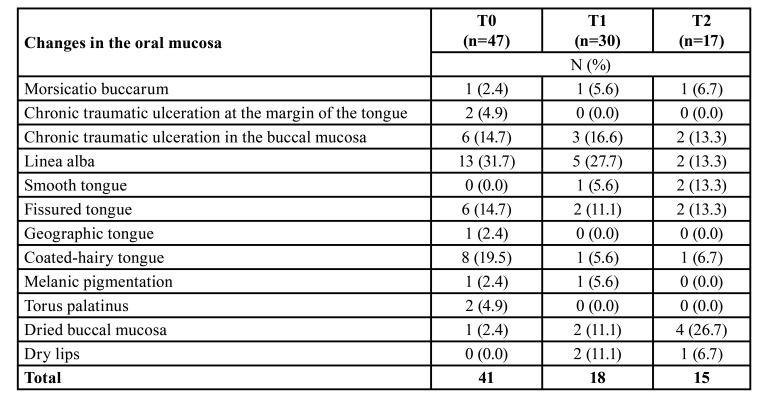
Description of the oral changes present in the study participants at the three time points (T0, T1, and T2).

**Table 3:**
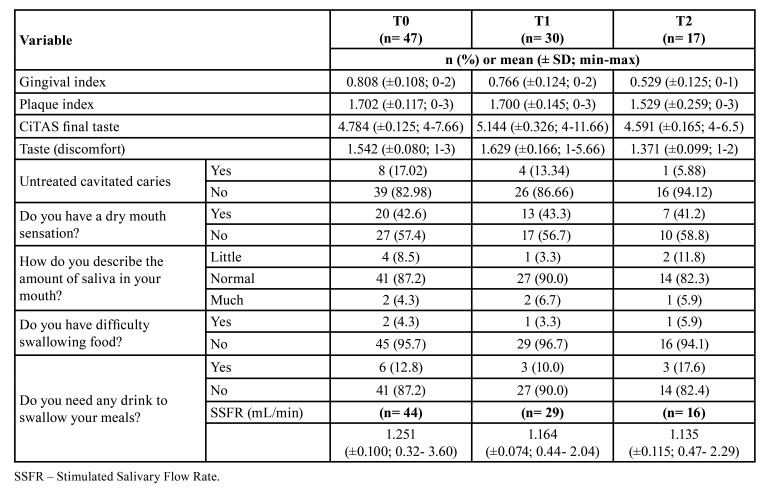
Mean values, standard deviation, minimum and maximum values of the gingival and plaque indices, taste, stimulated salivary flow rate, prevalence of untreated cavitated caries, and frequency of answers to questions about xerostomia.

**Table 4:**
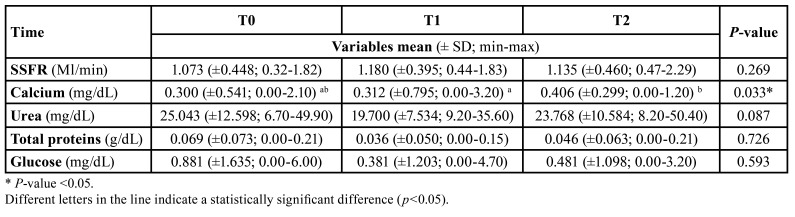
Mean, minimum and maximum values, standard deviation, and *p-value* of the stimulated salivary flow rate and salivary concentrations of calcium, urea, total proteins, and glucose of the participants evaluated at the three time points (T0, T1, and T2) (n = 16).

At T1, there was a significant correlation between “How do you describe the amount of saliva in your mouth” and taste (final CiTAS; *p*=0.039 r=-0.378) and “How do you describe the amount of saliva in your mouth” and taste (discomfort; *p*=0.027 r=-0.404).

At T2, there was a significant difference between the average score of taste (discomfort) in relation to the question "Do you need any drink to swallow meals?" and those who needed a drink to swallow meals had greater discomfort for taste (*p*=0.015). In T2, there was also a significant difference between the mean value of SSFR and the question, "Do you need a drink to swallow your meals?" and those who needed a drink to swallow their meals had a lower SSFR (*p*=0.026).

In T2, there was a significant regular and negative correlation between the SSFR values and taste (discomfort; *p*=0.037, r=-0.524).

## Discussion

Due to the limited number of studies on the adverse oral effects of PrEP and knowing that prolonged use of PrEP can cause systemic side effects (8,9), we aimed to assess the oral and saliva condition of HIV-uninfected people at risk of exposure to HIV and eligible for the use of PrEP, before starting the medication and at two further timepoints, over approximately 130 days. In the present sample of PrEP users, there was no difference between the oral and salivary conditions analyzed over time, except for the salivary calcium concentration that increased after the third medication dispensation compared to the second dispensation. This increase in calcium concentration may be related to bone remodeling caused by tenofovir (9), resulting in higher serum levels and salivary calcium.

Although there was no difference in the SSFR values of PrEP users among the three time points (T0, T1, T2), 36% (T1), and 28.7% (T2) of the changes felt by participants after beginning PrEP was dryness of their lips or oral mucosa, and, on physical examination, 22.2% (T1) and 33.4% (T2) of the changes were related to dried lips or mucosa. In addition, PrEP users who had dry mouth sensation (T1) or needed a drink to swallow meals (T2) had lower mean SSFR values. These data show the importance of reports of dry mouth, which may be associated with decreased salivary flow (12). Xerostomia is the subjective experience of dry mouth, and the diagnosis is based on asking patients whether they have any dry mouth symptoms. The causes of xerostomia go beyond inadequate salivary volume and include changes in salivary composition and the use of medications, such as antiretroviral drugs (10,22).

In the present study, the presence of xerostomia and “little” responses to the question “How do you describe the amount of saliva in your mouth?” was related to the worsening of taste at T1. In T1, PrEP users who needed a drink to swallow their meals and had a lower SSFR also reported worsened taste (discomfort). Saliva is essential for taste, as it helps in the transduction of flavoring, solubilizing, and facilitating movement to the taste pore, thereby binding to the receiving cells (23). Taste disorders, particularly dysgeusia and bitter taste, are common in users of protease inhibitor class antiretrovirals (10). These antiretrovirals can produce changes in taste through the secretion of the medication in the saliva or by diffusion of the lingual blood vessels to the basolateral face of the taste cells. With continuous use, the medication may be present in the taste buds, generating an accumulation of its metabolites, resulting in continued taste change (24).

Changes in salivary composition, such as increased calcium concentration, can contribute to the feeling of dry mouth, despite an adequate amount of saliva secretion (22,25). Also, dry mouth has been associated with changes in the salivary calcium concentration, generalized oral discomfort, and dysgeusia (22). Changes in taste are among the oral manifestations of HIV-infected patients, and the significant loss of taste perception is compounded by antiretroviral therapy (26).

In the saliva of PrEP users, the calcium concentration increased with longer medication use. Calcium metabolism is regulated by three main mechanisms: intestinal absorption, renal reabsorption, and bone remodeling. These, in turn, are regulated by a set of interacting hormones, including parathyroid hormone (PTH), 1,25-dihydroxy vitamin D [1,25 (OH) 2D], ionized calcium, and its corresponding receptors in the intestine, kidneys, and bones (27). Truvada® is a type of medicine known as an HIV-1 nucleoside analog reverse transcriptase inhibitor. It contains two active ingredients: emtricitabine and TDF, combined in a single coated Tablet. An *in vitro* study showed that tenofovir has a direct dose-dependent effect on the calcium-sensing receptor (CaSR), which may be responsible for the metabolic changes seen in patients receiving antiretroviral treatment (28). The elevated serum PTH concentration recorded in patients using TDF can be sustained by CaSR inhibitory activity, a receptor coupled to the class C protein G that detects extracellular levels of calcium ions, expressed mainly in the parathyroid gland and tubules kidney (28). Thus, by inactivating CaSR, TDF does not induce the stimulation of kinase activity regulated by an extracellular signal (ERK1/2) in parathyroid cells, leading to increased secretion of PTH (29) and consequently calcium. The initiation of antiretroviral treatment, including tenofovir/emtricitabine, is associated with an increase in plasma PTH concentrations shortly after the drug is introduced in the 4th, 24th, and 36th weeks of use (30). Consequently, hormonal changes, such as increased PTH, cause changes in calcium metabolism resulting in higher salivary calcium levels (24).

The participants in this study were mostly male, white, single, with complete or incomplete higher education, and had a satisfactory oral condition. Almost all of the participants reported access to dental care (97.8%). The sample was entirely composed of patients belonging to a key population at risk of exposure to HIV (1). These key populations are subject to disproportionate risks and vulnerabilities, do not have equiTable access to health care, experience health outcomes below those recommended, and deserve a priority response based on basic human rights (5). PrEP has emerged as an effective option for HIV prevention in these most vulnerable populations. However, its efficacy is directly related to a patient's adherence to treatment. Some factors that influence adherence to PrEP are stigma, low-risk perception, low decision-making power, an unaccepTable dosing regime, side effects, and the logistics of everyday life (5). Furthermore, adherence is better during times of higher perceived risk, although on average adherence to PrEP wanes over time (5). In the present study, low adherence to treatment over time was characterized as the main limitation and was evidenced by the loss of 30 patients over an average follow-up period of 130 days. The presence of systemic adverse effects of PrEP, such as nausea, malaise, diarrhea, vomiting, dizziness, and abdominal discomfort, may have contributed to the patients' loss of adherence in our study, as 44% of the changes felt in T1 and about 28.7% of the changes felt in T2 reported such effects. Thereafter, knowing that the sample selection was for convenience and that there were several losses, the level of reproducibility of the present study can be assured by the sample power superior to 75% performed from variables that showed statistically significant differences during follow-up.

Although our study was preliminary and the sample was limited, the results showed that the prolonged use of PrEP seems to be associated with reports of dry mouth and worsening of taste. These symptoms are possibly related to their increased salivary calcium concentrations associated with PrEP use. Our findings can help guide strategies that aim to minimize the impact of these symptoms on patients' quality of life. As few studies have been conducted on the adverse oral effects of PrEP, we were unable to make clear comparisons with previous studies. Further studies addressing this issue in chronic PrEP users should be conducted so that strategies aimed at the prevention and care of oral conditions can be formulated to improve the oral health-related quality of life of patients taking PrEP.
